# Prevalence and associated factors of structural congenital anomalies in resource limited setting, 2023: a systematic review and meta-analysis

**DOI:** 10.3389/fped.2023.1146384

**Published:** 2023-11-10

**Authors:** Yohannes Fikadu Geda, Yirgalem Yosef Lamiso, Tamirat Melis Berhe, Samuel Ejeta Chibsa, Tadesse Sahle, Kenzudin Assefa, Seid Jemal Mohammed, Seblework Abeje, Molalegn Mesele Gesese

**Affiliations:** ^1^ Department of Midwifery, Wolkite University, Wolkite, Ethiopia; ^2^ Department of Public Health, Wolkite University, Wolkite, Ethiopia; ^3^ Department of Midwifery, Mettu University, Mettu, Ethiopia; ^4^ Department of Nursing, Wolkite University, Wolkite, Ethiopia; ^5^ Department of Biochemistry, Wolkite University, Wolkite, Ethiopia; ^6^ Department of Public Health, Wolaita Sodo University, Wolaita Sodo, Ethiopia

**Keywords:** birth defect, congenital abnormalities, congenital anomalies, risk factor, resource-limited setting

## Abstract

**Background:**

Several studies have been conducted on structural congenital anomalies (CA). However, there is a paucity of studies that provide a comprehensive review of structural anomalies. We aimed to verify the available research articles to pool the possible risk factors of structural CA in resource-limited settings.

**Setting:**

The research articles were genuinely searched using PubMed, Scopus, Cochrane Library, Web of Science, free Google database search engines, Google Scholar, and ScienceDirect databases. Published studies were searched and screened for inclusion in the final analysis, and studies without sound methodologies and review and meta-analysis were not included in the analysis.

**Participants:**

This review analyzed data from 95,755 women who gave birth as reported by primary studies. Ten articles were included in this systematic review and meta-analysis. The articles that had incomplete information and case reports were excluded from the study.

**Results:**

The overall pooled effect estimate (EI) of structural CA was 5.50 (4.88–6.12) per 100 births. In this systematic review and meta-analysis, maternal illness EI with odds ratio (OR) = 4.93 (95% CI: 1.02–8.85), unidentified drug use with OR = 2.83 (95% CI: 1.19–4.46), birth weight with OR = 4.20 (95% CI: 2.12–6.28), chewing chat with OR = 3.73 (95% CI: 1.20–6.30), chemical exposure with OR = 4.27 (95% CI: 1.19–8.44), and taking folic acid tablet during pregnancy with OR = 6.01 (95% CI: 2.87–14.89) were statistically significant in this meta-regression.

**Conclusions:**

The overall pooled effect estimate of structural CA in a resource-limited setting was high compared to that in countries with better resources. Maternal illness, unidentified drug use, birth weight, chewing chat, chemical exposure, and never using folic acid were found to be statistically significant variables in the meta-regression. Preconception care and adequate intake of folic acid before and during early pregnancy should be advised.

**Systematic Review Registration:**

https://www.crd.york.ac.uk/PROSPERO/, identifier CRD42022384838.

## 1. Plain English summary

There are several primary studies conducted on the possible risk factors of structural congenital anomalies (CA) in resource-limited settings. However, no study shows the pooled effect of structural CA in resource-limited settings. Therefore, this study was designed to verify the best available articles to pool the possible risk factors of structural CA.

This study brings scientific information important for program planners, other researchers, and policy developers to have a qualified service delivery. In addition, it is also used by health professionals in using evidence-based practices to provide services.

Published and unpublished primary studies were included in this study irrespective of publication or study year. Variables statistically significant at least in two primary studies were screened using an Excel sheet. Meta-regression was conducted for all screened variables using the STATA version 14. 0 software.

The overall pooled effect estimate of structural CA in a resource-limited setting was high compared to that in countries with better resources. Maternal illness, unidentified drug use, birth weight, chewing chat, chemical exposure, and never using folic acid were found to be statistically significant variables in the meta-regression, which might be the possible risk factors of CA in low-resource settings.

Therefore, health officials in all resource-limited settings should advise women with illnesses like diabetes mellitus to have preconception care and antenatal care contact. In addition, health professionals should be strict when providing medications during pregnancy. They should check for possible teratogenicity of the drug before prescription and guidelines should be available at each care delivery office. Moreover, governments in resource-limited settings should advise on preconception care, vaccination, and adequate intake of folic acid before and during early pregnancy.

## 2. Background

CA, also known as birth defects, genetic disorders, or congenital malformations, imply functional or structural alterations that have a prenatal origin, and we can differentiate them in the perinatal period, neonatal period, or even years after birth (1, 2). We can classify CA as primary or minor depending on the magnitude of the structural and functional disorders and the need for medical support or treatment (1, 3, 4).

Depending on the phases of development at which the harm has occurred, congenital deformity can damage several organs (5, 6). Some studies have reported that central nervous system anomalies are the most predominant congenital malformations (5, 6). Heart and neural tube defects and Down syndrome represent the most common severe CA (1, 7).

Approximately 50% of CA have no defined cause; however, some genetic conditions, environmental agents, and infectious agents are known risk factors (8, 9). Approximately 2%–4% are attributed to parental chromosomal abnormalities; anatomical abnormality contributes to 10%–15%, endocrine factors contribute to 17%–20%, and antiphospholipid antibody syndrome contributes to 15%–20% (10). Many of the known causes of CA can be prevented with vaccination and the provision of proper antenatal care services during pregnancy (8, 11, 12).

Globally, it is estimated that about 7.9 million (6%) children were born with CA (1, 2). The World Health Organization (WHO) reported that about 17%–42% of infant mortality was attributed to CA (13) and, for the period 2000–2016, about 295,000 children died in the first 28 days after birth (14).

CA were the fifth leading cause of death for children under 5 years old and responsible for over 10% of all deaths in this age group (15). An estimated 94% of CA (16) and 96% of deaths due to CA occur in low-income countries (15).

In sub-Saharan Africa, approximately 10% of deaths among children under 5 years old are related to CA (15). Between 2.8% and 15.9% of Nigerians are reported to have congenital abnormalities (5) and from 0.9% to 17.3% in Ethiopia (17–19).

CA reported in Ethiopia include anencephaly, hydrocephalus, spina bifida, meningomyelocele, umbilical hernia, orofacial, neural tube, upper and lower limb, cardiovascular system, digestive system, abdominal wall, unspecified congenital malformations, Down syndrome, genitourinary system, cleft lip and palate, clubfoot, hernias, and head, face, and neck defects (17–20).

Sociodemographic characteristics such as maternal age, women living in urban, educational status, nutritional status, intake of herbal and unprescribed medicine, folic acid supplementation status, drinking alcohol, and occupational status were the determinants for CA (21–23). The prognosis for a favorable pregnancy outcome is normally about 80% if contributory factors of CA are identified and treated (24). CA can be treated with surgical and non-surgical options unless, otherwise, these can cause lifelong impacts (20). Despite this, CA have received less attention in low-resource settings, which has led to a large gap in knowledge and understanding about their prevalence and risk factors (15, 22, 23).

Even though there have been some primary articles conducted on possible risk factors of structural CA in resource-limited settings, there is no study that serves as a reference for these settings. Therefore, this systematic review and meta-analysis is designed to verify the available articles to pool the possible risk factors of structural CA in resource-limited settings. The result and conclusion of this study will give program planners, other researchers, and policymakers scientific knowledge to help them enhance service delivery. Furthermore, it will be useful for health professionals in using evidence-based practices to provide the services.

## 3. Methods

### 3.1. Study design and setting

The authors assessed the PROSPERO database (https://www.crd.york.ac.uk/PROSPERO/) for all published or ongoing research available related to the title to avoid any further duplication. Accordingly, the result showed that there were no ongoing or published articles in the area of this title. Therefore, this review and meta-analysis was registered in the PROSPERO database with an identification number of CRD42022384838 on 28 December 2022. This review and meta-analysis was conducted to verify the pooled possible risk factors of structural CA in resource-limited settings. Scientific consistency was formulated by using the preferred reporting items of systematic reviews and meta-analysis (PRISMA) checklist (25).

### 3.2. Information source

A systematic and genuine search of the research articles was done via the following listed databases: PubMed, Scopus, Cochrane Library, Web of Science, free Google database search engines, Google Scholar, and ScienceDirect search engines. We have used the keywords (((((((Congenital Abnormalities/abnormalities) OR Congenital Anomalies/anomalies [MeSH Terms]) OR Birth Defects/defects [MeSH Terms]) AND Risk Factors) OR Associated factors [MeSH Terms]) OR Influencing factors [MeSH Terms]) AND Each low-income country name) OR Resource-Limited Settings [MeSH Terms: No Exp].

The search was performed using the following key search terms: “AND” and “OR” Boolean operators individually and in combination with each other. Moreover, the reference lists of all the included studies were also searched to identify any other studies that may have been missed by the search strategy. The search for all research was done from 10 October 2022 to 5 December 2022 without limiting the publication dates of the literature.

### 3.3. Eligibility criteria

Published articles in national and international journals in resource-limited settings with a result of possible risk factors of structural CA were included in this study. They were searched and screened for inclusion in the final analysis. This study included available observational study designs (cross-sectional studies and case–control studies). All research articles that were published and accessed from the repositories till the final date of data analysis and submission of this manuscript to this journal were included in accordance with these criteria.

**Figure 1:**
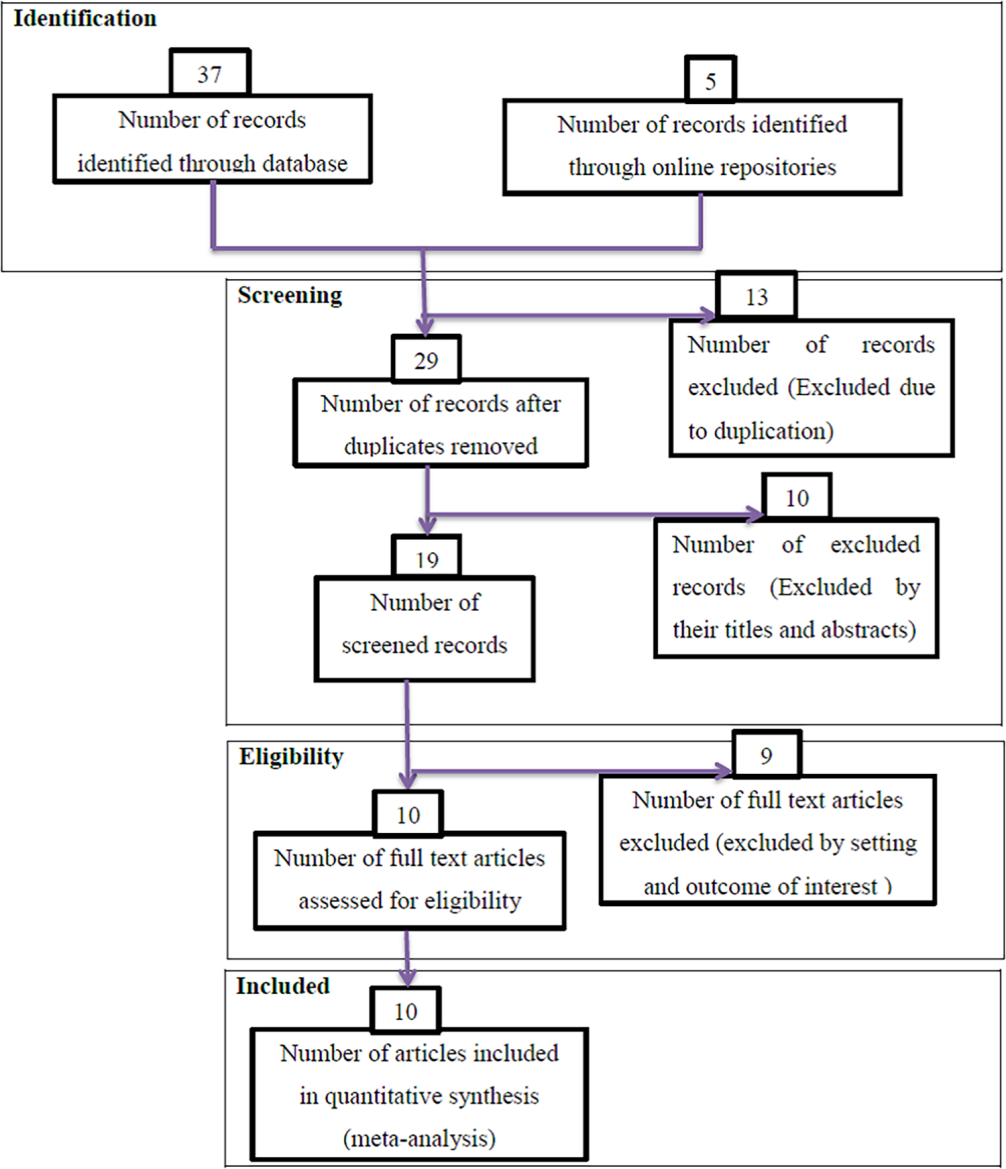
PRISMA flow diagrams of included studies in the systematic review and meta-analysis on possible risk factors of congenital anomalies in a resource-limited setting, 2022.

During the beginning of our search, 42 studies were found, of which 13 were skipped due to duplication and 29 were identified for eligibility. Of the 29 studies, 10 were excluded from the highlight review on their abstracts, and 19 studies were assessed for full text. Of these 19 studies, nine were excluded due to non-relevance to the current review, and 10 were included in the final meta-analysis of this study (Figure 1).

Studies without sound methodologies and review and meta-analysis were not included in this analysis. Articles without full information that is important for the analysis and case reports were excluded from the study. Duplication of results in studies and outcome variable measures with inconsistency were excluded from the final analysis. Studies that incorporate other types of CA were excluded (Figure 1).

### 3.4. Operational definition

**Structural CA** are structural changes, whether substantial or slight, that have a significant impact on the health or appearance of an individual and often demand medical attention (26).

**Resource-limited settings** are categorized as low-income nations by the World Bank, a global alliance of nations devoted to eradicating poverty, which determined that they had the weakest economy (27).

### 3.5. Quality assessment and data extraction

The basic quality of the included research articles was evaluated using the Newcastle–Ottawa scale (NOS). The NOS was created to evaluate the quality of observational research articles in systematic reviews and meta-analyses. Data from this study were extracted by the two authors (YG and YL) using a standardized data extraction checklist on an Excel sheet.

This systematic review and meta-analysis uses a PRISMA flowchart to differentiate and pick important articles for the analysis. During commencement, duplicated types of studies were not included by using the EndNote version X8.1 referencing tool. The articles were excluded by adding highlight reviewing to their titles and abstracts before assessing the whole text. Full-text studies or research results were assessed for the rest studies. Depending on the stated eligibility criteria above, the eligibility of the articles was evaluated.

Data were extracted using the standardized data extraction tool in considering the name of the first author, publication year, study setting, target population, study area, study design, sample size, the status of structural CA, and associated factors risk estimate (OR) and their 95% confidence interval (CI) (Table 1). All pieces of literature were checked by the two authors independently (YG and YL). When there were disagreements, the articles were further reviewed by one of the authors (TB) and used as final mediation and eligibility decisions.

**Table 1:** Descriptive summary of included articles to pool possible risk factors of congenital anomalies in a low-resource setting, 2022.

Authors	Year	Design	Study area	Sample size	Number of cases	Inclusion criteria of cases
Abebe et al. (28)	2021	Case–control	Southwestern Ethiopia	1,138	251	Live birth or fresh stillbirth
Bekalu et al. (29)	2019	Cross-sectional	Jimma	754	31	Total births with CA
Eshete et al. (30)	2020	Case–control	Addis Ababa	156,272	3,215	Total births with CA
Feredegn et al. (31)	2018	Cross-sectional	Addis Ababa	271	97	Live births
Gedamu et al. (32)	2021	Cross-sectional	Bishoftu	2,218	23	Live births
Jemal et al. (33)	2021	Case–control	Arsi	418	105	Total births externally visible defects
Mekonen et al. (27)	2021	Cross-sectional	Bahir Dar	11,177	69	Total births with CA
Musa et al. (34)	2020	Cross-sectional	Addis Ababa	116	71	Live births
Sileshi et al. (35)	2021	Cross-sectional	Jimma	3,346	199	Live births
Taye et al. (36)	2019	Cross-sectional	Addis Ababa and Amhara	76,201	1,518	Live births

### 3.6. Data synthesis and analysis

Both systematic review and meta-analysis were conducted, and the software used for the analysis was the STATA version 14.0 software. Data were imported into STATA for additional analysis after being extracted using a standardized extraction method. All necessary analyses were performed using the software's metan commands, and the results of those analyses were interpreted accordingly. Quantitative reviews were conducted to determine the overall pooled possible risk factors of structural CA in low-resource settings. The degree of heterogeneity between the included studies was evaluated by determining the *p*-values of *I*^2^ test statistics. *I*^2^ test statistics scores of 0%, 25%, 50%, and 75% were taken as no, low, moderate, and high degrees of heterogeneity, respectively (32). Due to the observed high heterogeneity across studies, we used a random-effect model to assess pooled estimates. Publication bias was checked by funnel plot. A *p*-value of less than 0.05 was used as the cutoff point for statistical significance of publication bias. Egger test was done and verified that there were no small-study effects.

## 4. Results

### 4.1. Selection and characterization of included studies

Ten articles were included in this systematic review and meta-analysis, and it was summarized in Table 1. Seven articles of the included study used a cross-sectional study design (29–31, 33–36), whereas three articles were case–control studies (28, 37, 38) with a sample size ranging from 418 in Arsi (28) to 76,201 in Addis Ababa and Amhara region (34).

In relation to the geographical location in which the study was conducted, four articles were from central Ethiopia (28, 31, 34, 37), one study from Northern Ethiopia (36), and three studies from southwestern Ethiopia (30, 33, 38) (Table 1). To obtain the pooled possible risk factor of CA, a random-effect model was used.

### 4.2. Publication bias

**Figure 2:**
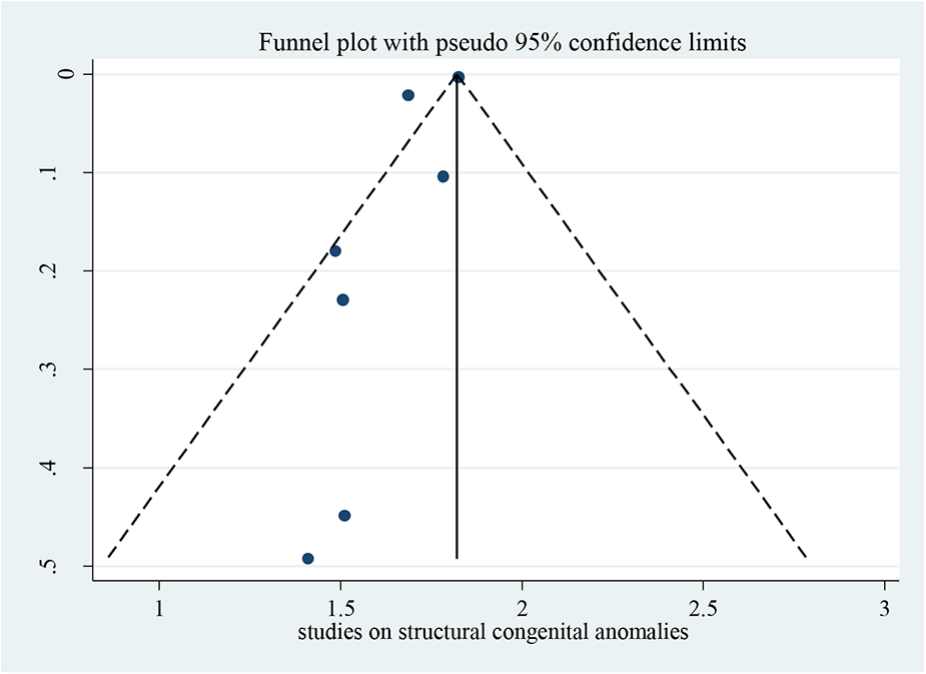
Funnel plot for studies on possible risk factors of congenital anomalies in a resource-limited setting, 2022.

Bias among the included studies was checked by the funnel plot at a 5% significance level. The funnel plot was symmetry and showed no statistical significance for the presence of publication bias for each variable. Egger test was done and verified that there were no small-study effects with *p* = 0.063 (Figure 2).

### 4.3. Structural CA

**Figure 3:**
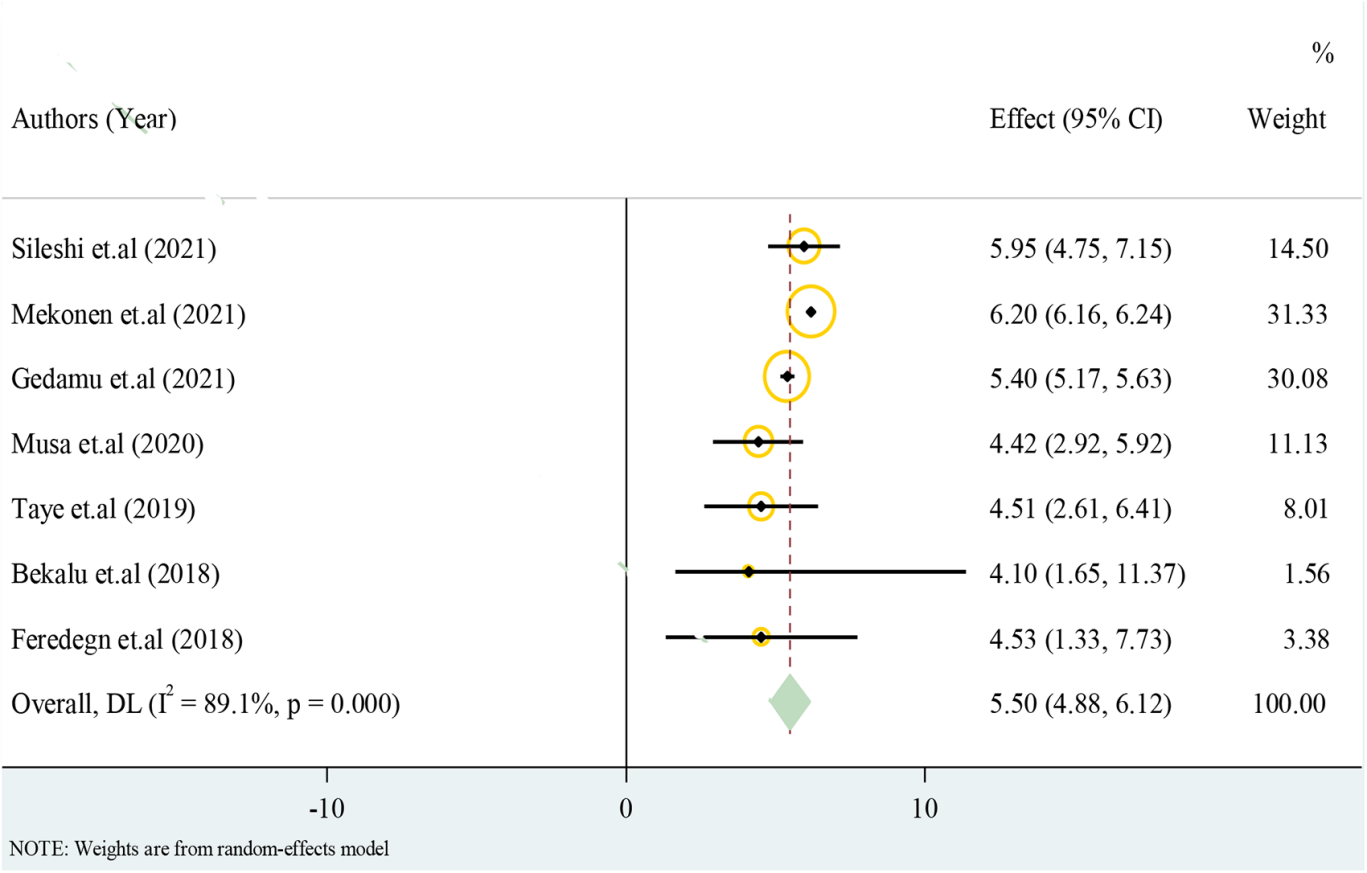
Forest plot for structural congenital anomalies in a resource-limited setting, 2022.

Only cross-sectional studies eligible for the analysis have reported the prevalence of structural CA. The overall pooled effect estimate of structural CA was 5.50 with a 95% CI of 4.88–6.12 from 100 births (Figure 3).

### 4.4. Possible risk factors of CA in a low-resource setting

In this systematic review and meta-analysis, previous history of abortion, maternal illness, history of alcohol intake during pregnancy, unidentified drug use, birth weight, chewing chat, chemical exposure, and taking folic acid tablets during pregnancy were statistically significant at one or more of the included primary studies. However, maternal illness, unidentified drug use, birth weight, chewing chat, chemical exposure, and taking folic acid tablets during pregnancy were staying statistically significant in this meta-regression.

This review analyzed data from 95,755 women who gave birth to estimate the pooled possible risk factors of CA in low-resource settings. A total of 10 (nine published and one unpublished) articles were included in this review (Table 1).

#### 4.4.1. Maternal illness

Meta-analysis pooling of aggregate data using the random-effect and inverse-variance model with Der Simonian–Laird estimate of tau^2^ was done for “maternal illness” separately. The test of the pooled overall effect provides 4.93 with a 95% CI: 1.02–8.85, which shows neonates of women with previous illness were 4.93 times more likely to have structural CA compared to women who have no history of illness (Table 2).

**Table 2:** Meta-regression result of pooled possible risk factors of congenital anomalies in a resource-limited setting, 2022.

Authors	Effect	95% CI	% weight
Maternal illness			
Jemal et al. (33)	6.10	2.39–15.57	35.23
Bekalu et al. (29)	4.30	1.65–11.37	64.77
Overall pooled	4.93	1.02–8.84	100.00
Unidentified drug use			
Abebe et al. (28)	3.4	2.0–5.8	42.77
Feredegn et al. (31)	2.2	1.1–4.0	56.35
Bekalu et al. (29)	15.1	5.5–40.2	0.88
Overall pooled	2.83	1.19–4.46	100
Birth weight < 2.5 kg			
Mekonen et al. (27)	4.56	2.76–7.55	75.55
Gedamu et al. (32)	3.10	1.23–9.65	24.45
Overall pooled	4.20	2.12–6.28	100.00
Chat chewing			
Bekalu et al. (29)	3.41	1.50–7.90	62.88
Jemal et al. (33)	4.76	1.57–14.47	15.48
Abebe et al. (28)	3.93	1.30–12.20	21.64
Overall pooled	3.73	1.20–6.30	100.00
Chemical exposure			
Jemal et al. (33)	4.76	1.57–14.47	41.70
Abebe et al. (28)	3.93	1.26–12.17	58.30
Overall pooled	4.27	1.19–8.44	100.00
Never use folic acid			
Jemal et al. (33)	0.57	0.41–0.73	25.00
Abebe et al. (28)	1.78	1.38–2.17	24.99
Bekalu et al. (29)	4.10	3.89–4.22	25.00
Gedamu et al. (32)	17.64	17.50–17.78	25.00
Overall pooled	6.01	2.87–14.89	100.00

#### 4.4.2. Unidentified drug use

Meta-regression of “unidentified drug use” with the data using the random-effect and inverse-variance model shows that unidentified drug use during pregnancy was significantly associated with CA in low-resource settings. Women who had a history of unidentified drug use during pregnancy were 2.83 times more likely to have structural CA compared to women who had no history of drug use during pregnancy (Table 2).

#### 4.4.3. Birth weight

Birth weight was found to be a statistically significant variable associated with structural CA in resource-limited settings. Neonates with a birth weight less than 2.5 kg were more likely to have structural CA compared to neonates with a birth weight greater than or equal to 2.5 kg. The meta-regression of birth weight considering random-effect and inverse-variance model had 4.2 overall effects with a 95% CI of 2.12–6.29 (Table 2).

#### 4.4.4. Chat chewing

Pregnant women who have chat chewing experience were found to have significant structural CA in the primary studies. The overall pooled effect women chewing chat were 3.73 times more likely to have structural CA compared to women who never chew chat (Table 2).

#### 4.4.5. Never use folic acid

Never using folic acid was a statistically significant variable in several primary studies and in the meta-regression as well. Pregnant women who did not use iron folate were 6.01 times more likely to have neonates with structural CA compared to those who used folic acid during and before pregnancy (Table 2).

#### 4.4.6. Subgroup analysis to pool possible risk factors of structural CA

**Figure 4:**
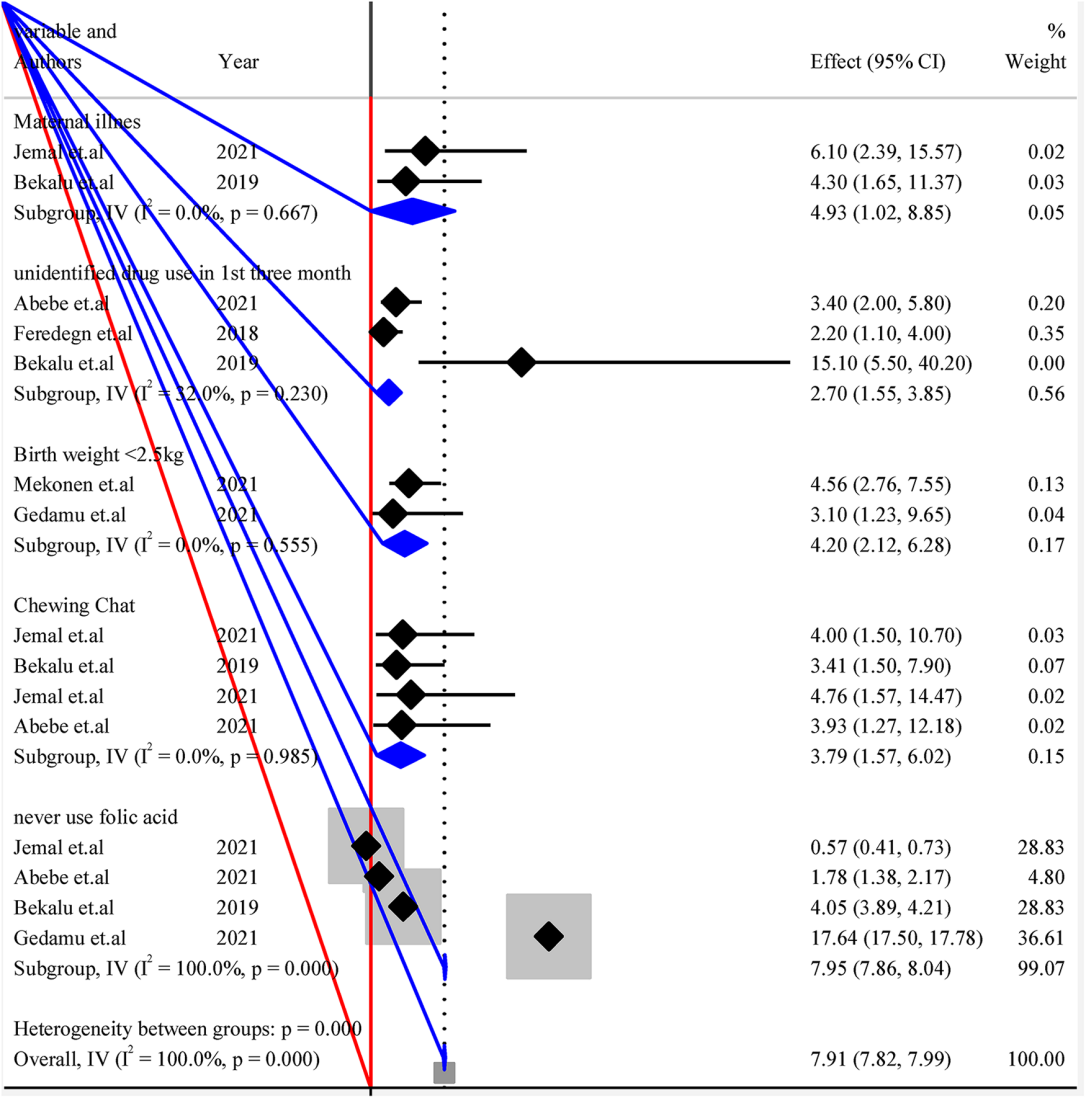
Forest plot of subgroup analysis by variables for pooled possible risk factors of congenital anomalies in a low-resource setting, 2022.

The listed individual variables were repeated in the analysis of a study within subgroups of subjects defined by a subgrouping variable. Each variable was presented with *I*^2^ and *p*-value to see the heterogeneities between studies (Figure 4).

## 5. Discussion

This study was initially planned to be conducted among countries with low-income economies in the current 2023 fiscal year according to the World Bank. Studies conducted in listed countries were extremely reviewed. Unfortunately, there was no research on the listed 28 low-income countries except Ethiopia, which fulfills the inclusion criteria. Those primary studies conducted in Ethiopia believed to nominate the low-resource setting of low-income countries till articles are available.

Published studies were included in this study irrespective of publication or study year. Variables statistically significant at least in two primary studies were screened using an Excel sheet. Meta-regression was conducted for all screened variables using the STATA version 14.0 software.

The overall pooled effect estimate of structural congenital anomalies in resource-limited settings was 5.50 with a 95% CI of 4.88–6.12. Maternal illness, unidentified drug use, birth weight, chewing chat chemical exposure, and never using folic acid were found to be statistically significant variables in the meta-regression, which might be the possible risk factors of CA in low-resource settings.

This study showed that maternal illness was one of the possible risk factors for CA in resource-limited settings. Consistently a study concluded that maternal exposure to illness, fever, and medication (particularly aspirin) may increase the risk of CA (39). Another study conducted on the association between CA and gestational diabetes mellitus stated that there was an increased rate of CA in the offspring of women with diabetes (40). A study reported that first-trimester maternal influenza exposure was associated with an increased risk of any CA (41). This might be due to the causative agent of such diseases that could pass the placental barrier and cause structural anomalies. However, experimental studies need to be conducted to confirm the associations.

This study verified that unidentified drug use was one of the possible risk factors for CA in resource-limited settings. Similarly, studies showed that first-trimester paroxetine, fluoxetine, sertraline, and anti-thyroid drug therapy exposures were associated with a significant increase in the risk of major CA (42–47).

This might be due to those drugs needing to be categorized as drugs that demonstrate fetal abnormalities. However, positive evidence of fetal risk in humans exists, but the benefit from use in pregnant women may be acceptable despite the risk, such as a life-threatening situation for which a safer agent cannot be used.

In this study birth weight was found to be significantly affected by CA in low-resource settings. In the same manner, studies reported that CA increased the risk of in-hospital mortality and was associated with short-term neonatal morbidities in low birth weight infants (48, 49). Another study states that the prevalence of neonates with low birth weight and CA was very high (50). This might be due to fetuses with structural anomalies having difficulties in using nutrients provided by the placenta appropriately due to their deformation. Moreover, fetuses with structural anomalies are more likely to have functional anomalies that might disrupt metabolism and growth in the uterus.

In this study, chewing chat was the possible risk factor for CA in low-resource settings. Similarly, a study conducted in Yemen clarifies that women who had chewed chat were more likely to have a poor neonatal outcome (51). This might show that chemicals in chat could pass the placental barrier and cause the anomalies. On the other hand, the consumption of chat affects the growth of the fetus by inhibiting blood flow from the uterus to the placenta, which in turn affects the normal growth of the fetus.

This study revealed that chemical exposure was a possible risk factor for CA in low-resource settings. Consistently, a study investigated the strong association between CA and exposure of mothers to air pollution by nitrogen dioxide during pregnancy by combining risk estimates for a variety of air pollutants (52). Another study reported evidence for an effect of ambient air pollutants on CA risk (53). This might show that chemicals in the work or living environment of pregnant women could cause structural CA. This may suggest that especially work environment of pregnant women needs to be screened for potential chemicals able to cause anomalies.

This study showed that clients who had never taken folic acid tablets were more likely to develop CA or clients who had taken folic acid tablets were less likely to develop CA compared to those who had not taken folic acid. Similarly, a study states that maternal preconception folic acid supplementation was significantly associated with the risk of CA (54). A study shows a robust estimate of the positive association between maternal folate supplementation and a decreased risk of CA (55). This might be due to the intake of folic acid prior to conception and during the early stages of pregnancy plays an important role in preventing structural CA.

## 6. Strength and limitation

This systematic review and meta-analysis brings a summative analysis of all primary studies conducted in resource-limited settings. All variables available in each article were assessed for significance in the pooled effect. The pooled possible risk factors of structural CA were obtained, and the pooled significant variables were identified. However, this systematic review might not be generalized to all countries with resource-limited settings. Studies carried out in settings with limited resources were thoroughly reviewed. Unfortunately, no research that satisfies the inclusion criteria has been carried out in settings other than Ethiopia. The primary research done in Ethiopia was assumed to nominate the weak resource conditions of low-income countries until articles were available.

## 7. Conclusion and recommendation

The overall pooled effect estimate of structural CA in a resource-limited setting was high compared to that in those countries with better resources. Maternal illness, unidentified drug use, birth weight, chewing chat, chemical exposure, and never using folic acid were found to be statistically significant variables in the meta-regression, which might be the possible risk factors of CA in low-resource settings.

Therefore, the following recommendations were given based on the obtained result of this study:

• Health officials in all resource-limited settings should advise women with illnesses like diabetes mellitus to have preconception care and antenatal care contact.
• Health professionals should be strict when providing medications during pregnancy. They should have to check for possible teratogenicity of the drug before prescription and guidelines should be available in each care delivery office.
• Prevention based on reproduction options includes teratogen information like chewing chat and chemical exposure, and prenatal screening for fetal anomalies should be done by all hospitals delivering preconception and pregnancy services.
• Governments in resource-limited settings should advise on preconception care and adequate intake of folic acid before and during early pregnancy.

## Data Availability

The data analyzed in this study is subject to the following licenses/restrictions: The data that support the findings of this study are available, but restrictions apply to the availability of these data, which were used under license for the current study, and so are not publicly available. Data are, however, available from the first author upon reasonable request. Requests to access these datasets should be directed to nechsar@gmail.com.

## References

[1] WHO/CDC/ICBDSR. Birth defects surveillance training: facilitator's guide. Geneva: World Health Organization (2016).

[2] World Health Organization. Zika situation report: neurological syndrome and congenital anomalies. (2016). https://iris.who.int/handle/10665/204348

[3] Bacino C. Birth defects: epidemiology, types, and patterns. UpToDate (2018). Available at: https://www.uptodate.com/contents/birth-defects-epidemiology-types-and-patterns (Accessed July 2, 2018).

[4] Boyle B Addor M-C Arriola L Barisic I. Estimating global burden of disease due to congenital anomaly. Arch Dis Child Fetal Neonatal Ed. (2018) 103(1):F22–8. 10.1136/archdischild-2016-311845 28667189 PMC5750368

[5] Obu HA Chinawa JM Uleanya ND Adimora GN Obi IE. Congenital malformations among newborns admitted in the neonatal unit of a tertiary hospital in Enugu, South-East Nigeria: a retrospective study. BMC Res Notes. (2012) 5:177. 10.1186/1756-0500-5-177 22472067 PMC3393607

[6] Ajao AE Adeoye I. Prevalence, risk factors and outcome of congenital anomalies among neonatal admissions in Ogbomoso, Nigeria. BMC Pediatr. (2019) 19(1):88. 10.1186/s12887-019-1471-1 30943931 PMC6446329

[7] Stallings EB Isenburg JL Short TD. Population-based birth defects data in the United States, 2012–2016. Birth Defects Res. (2019) 111(18):1436–47. 10.1002/bdr2.1607 31642616 PMC6886260

[8] Lee KS Choi YJ Cho J. Environmental and genetic risk factors of congenital anomalies: an umbrella review of systematic reviews and meta-analyses. J Korean Med Sci. (2021) 36(28):e183. 10.3346/jkms.2021.36.e183 34282604 PMC8289720

[9] WHO. Congenital anomalies. World Health Organization (2018). Available at: http://www.who.int/news-room/fact-sheets/detail/congenital-anomalies. (Accessed December 28, 2022)

[10] UN. European monitoring of congenital anomalies (2020). Available at: https://op.europa.eu/en/publication-detail/-/publication/cc2ffa13-d854-11ea-adf7-01aa75ed71a1/language-en. (Accessed December 28, 2022)

[11] Tsehay B Shitie D Lake A Abebaw E. Determinants and seasonality of major structural birth defects among newborns delivered at primary and referral hospital of East and West Gojjam zones, Northwest Ethiopia. BMC Res Notes. (2019) 12(1):495. 10.1186/s13104-019-4541-4 31399144 PMC6688374

[12] Mendes IC Amorim Jesuino RS da Silva Pinheiro D Silva Rebelo AC. Anomalias congênitas e suas principais causas evitáveis: uma revisão. Rev méd Minas Gerais. (2018). 10.5935/2238-3182.20180011

[13] Boyle B Addor M-C Arriola L Barisic I Bianchi F. Estimating global burden of disease due to congenital anomaly: an analysis of European data. BMJ Open. (2018). 10.1136/archdischild-2016-311845 PMC5750368 28667189

[14] França E Lansky S Rego MA Malta D França JS Teixeira R Leading causes of child mortality in Brazil, in 1990 and 2015: estimates from the global burden of disease study. Rev Bras Epidemiol. (2017). 10.1186/s12963-017-0156-y 28658372

[15] UKRI. Sub-Saharan African Network for congenital anomalies: surveillance, prevention and care (2022). Available at: https://gtr.ukri.org/projects?ref=MR%2FT039132%2F1#/tabOverview. (Accessed December 28, 2022)

[16] WHO. Congenital anomalies (2022). Available at: https://www.who.int/teams/integrated-health-services/clinical-services-and-systems/surgical-care/congenital-anomalies. (Accessed December 28, 2022)

[17] Geneti SA Dimsu GG Sori DA. Prevalence and patterns of birth defects among newborns in southwestern Ethiopia: a retrospective study. Pan Afr Med J. (2021) 40:248. 10.11604/pamj.2021.40.248.25286 35233268 PMC8831222

[18] Mekonnen D MollaTaye Worku W. Congenital anomalies among newborn babies in Felege-Hiwot Comprehensive Specialized Referral Hospital, Bahir Dar, Ethiopia. Sci Rep. (2021) 11(1):11027. 10.1038/s41598-021-90387-0 34040058 PMC8154920

[19] Taye M Afework M Fantaye W. Magnitude of birth defects in Central and Northwest Ethiopia from 2010 to 2014: a descriptive retrospective study. PLoS One. (2016) 11(10):e0161998. 10.1371/journal.pone.0161998 27706169 PMC5051902

[20] WHO. Congenital anomalies (2021). Available at: https://www.who.int/health-topics/congenital-anomalies#tab=tab_1. (Accessed December 28, 2022)

[21] Tsehay B Shitie D Lake A. Determinants and seasonality of major structural birth defects among newborns delivered at primary and referral hospital of East and West Gojjam zones, Northwest Ethiopia 2017–2018: case–control study. BMC Res Notes. (2019) 12(1):495. 10.1186/s13104-019-4541-4 31399144 PMC6688374

[22] Sitkin NA Ozgediz D Donkor P Farmer DL. Congenital anomalies in low- and middle-income countries: the unborn child of global surgery. World J Surg. (2015) 39(1):36–40. 10.1007/s00268-014-2714-9 25135175 PMC4300430

[23] Ajao AE Adeoye IA. Prevalence, risk factors and outcome of congenital anomalies among neonatal admissions in OGBOMOSO, Nigeria. BMC Pediatr. (2019) 19(1):88. 10.1186/s12887-019-1471-1 30943931 PMC6446329

[24] Singh G Sidhu K. Bad obstetric history a prospective study. Med J Armed Forces India. (2010) 66(2):117–20. 10.1016/S0377-1237(10)80121-2 27365723 PMC4920907

[25] Page MJ McKenzie JE Bossuyt PM Boutron I Hoffmann TC Mulrow CD The PRISMA 2020 statement: an updated guideline for reporting systematic reviews. PLoS Med. (2021) 18(3):e1003583. 10.1371/journal.pmed.1003583 33780438 PMC8007028

[26] WHO/CDC/ICBDSR. Birth defects surveillance training: facilitator’s guide. Geneva: World Health Organization (2015).

[27] World Bank. New World Bank country classifications by income level: 2022–2023 (2023). Avialble at: https://blogs.worldbank.org/opendata/new-world-bank-country-classifications-income-level-2022-2023.

[28] Jemal S Fentahun E Oumer M Muche A. Predictors of congenital anomalies among newborns in Arsi zone public hospitals, Southeast Ethiopia: a case-control study. Ital J Pediatr. (2021) 47(1):143. 10.1186/s13052-021-01093-6 34193221 PMC8243734

[29] Musa AH Afework M Bedru M Tamirat S. Incidence of atrial septal defects in children attended the Cardiac Center of Ethiopia during January 2016 to December 2018. bioRxiv preprint (2020).

[30] Getachew B. Prevalence of overt congenital anomalies, and associated factors among newborns delivered at Jimma University Medical Center, southwest Ethiopia. Ethiopia: Jimma University (2018).

[31] Gedamu S Sendo EG Daba W. Congenital anomalies and associated factors among newborns in Bishoftu General Hospital, Oromia, Ethiopia: a retrospective study. J Environ Public Health. (2021) 2021:2426891. 10.1155/2021/2426891 33859704 PMC8026314

[32] Higgins JP Thompson SG. Quantifying heterogeneity in a meta-analysis. Stat Med. (2002) 21(11):1539–58. 10.1002/sim.1186 12111919

[33] Silesh M Lemma T Fenta B Biyazin T. Prevalence and trends of congenital anomalies among neonates at Jimma Medical Center, Jimma, Ethiopia: a three-year retrospective study. Pediatric Health Med Ther. (2021) 12:61–7. 10.2147/PHMT.S293285 33628075 PMC7898197

[34] Taye M Afework M Fantaye W Diro E Worku A. Congenital anomalies prevalence in Addis Ababa and the Amhara region, Ethiopia: a descriptive cross-sectional study. BMC Pediatr. (2019) 19(1):234. 10.1186/s12887-019-1596-2 31296186 PMC6625051

[35] Talarge F Seyoum G Tamirat M. Congenital heart defects and associated factors in children with congenital anomalies. Ethiop Med J. (2018) 56(4). 10.1016/j.ijans.2022.100513

[36] Mekonnen DM Worku W. Congenital anomalies among newborn babies in Felege-Hiwot Comprehensive Specialized Referral Hospital, Bahir Dar, Ethiopia. Sci Rep. (2021) 11(1):11027. 10.1038/s41598-021-90387-0 34040058 PMC8154920

[37] Eshete M Abate F Abera B Hailu A Demissie Y Mossey P Assessing the practice of birth defect registration at Addis Ababa health facilities. Ethiop J Health Sci. (2021) 31(3):683–7. 10.4314/ejhs.v31i3.26 34483626 PMC8365477

[38] Abebe S Gebru G Amenu D Mekonnen Z Dube L. Risk factors associated with congenital anomalies among newborns in southwestern Ethiopia: a case-control study. PLoS One. (2021) 16(1):e0245915. 10.1371/journal.pone.0245915 33508017 PMC7843017

[39] Abe K Honein MA Moore CA. Maternal febrile illnesses, medication use, and the risk of congenital renal anomalies. Birth Defect. (2003) 67(11):911–18. 10.1002/bdra.10130 14745928

[40] Zhang TN Huang XM Zhao XY Wang W Wen R Gao SY. Risks of specific congenital anomalies in offspring of women with diabetes: a systematic review and meta-analysis of population-based studies including over 80 million births. PLoS Med. (2022) 19(2):e1003900. 10.1371/journal.pmed.1003900 35104296 PMC8806075

[41] Luteijn JM Brown MJ Dolk H. Influenza and congenital anomalies: a systematic review and meta-analysis. Hum Reprod. (2014) 29(4):809–23. 10.1093/humrep/det455 24365800

[42] Uguz F. Selective serotonin reuptake inhibitors and the risk of congenital anomalies: a systematic review of current meta-analyses. Expert Opin Drug Saf. (2020) 19(12):1595–604. 10.1080/14740338.2020.1832080 33001713

[43] Bar-Oz B Einarson T Einarson A Boskovic R O'Brien L Malm H Paroxetine and congenital malformations: meta-analysis and consideration of potential confounding factors. Clin Ther. (2007) 29(5):918–26. 10.1016/j.clinthera.2007.05.003 17697910

[44] Agrawal M Lewis S Premawardhana L Dayan CM Taylor PN Okosieme OE. Antithyroid drug therapy in pregnancy and risk of congenital anomalies: systematic review and meta-analysis. Clin Endocrinol (Oxf). (2022) 96(6):857–68. 10.1111/cen.14646 34845757

[45] Shen ZQ Gao SY Li SX Zhang TN Liu CX Lv HC Sertraline use in the first trimester and risk of congenital anomalies: a systemic review and meta-analysis of cohort studies. Br J Clin Pharmacol. (2017) 83(4):909–22. 10.1111/bcp.13161 27770542 PMC5346877

[46] Li H Zheng J Luo J Zeng R Feng N Zhu N Congenital anomalies in children exposed to antithyroid drugs in-utero: a meta-analysis of cohort studies. PLoS One. (2015) 10(5):e0126610. 10.1371/journal.pone.0126610 25974033 PMC4431808

[47] Yakoob MY Bateman BT Ho E Hernandez-Diaz S Franklin JM Goodman JE The risk of congenital malformations associated with exposure to beta-blockers early in pregnancy: a meta-analysis. Hypertension. (2013) 62(2):375–81. 10.1161/HYPERTENSIONAHA.111.00833 23753416 PMC4086784

[48] Chung SH Kim CY Lee BS. Congenital anomalies in very-low-birth-weight infants: a nationwide cohort study. Neonatology. (2020) 117(5):584–91. 10.1159/000509117 32772029

[49] Venetis CA Papadopoulos SP Campo R Gordts S Tarlatzis BC Grimbizis GF. Clinical implications of congenital uterine anomalies: a meta-analysis of comparative studies. Reprod Biomed Online. (2014) 29(6):665–83. 10.1016/j.rbmo.2014.09.006 25444500

[50] Mekonen HK Nigatu B Lamers WH. Birth weight by gestational age and congenital malformations in northern Ethiopia. BMC Pregnancy Childbirth. (2015) 15:76. 10.1186/s12884-015-0507-2 25886401 PMC4381366

[51] Abdel-Aleem AM Abdulbari Assad MM. Khat chewing during pregnancy: an insight on an ancient problem. Impact of chewing khat on maternal and fetal outcome among Yemeni pregnant women. J Gynecol Neonatal Biol. (2015) 1(2):28. 10.15436/2380-5595.15.004

[52] Chen EK Zmirou-Navier D Padilla C Deguen S. Effects of air pollution on the risk of congenital anomalies: a systematic review and meta-analysis. Int J Environ Res Public Health. (2014) 11(8):7642–68. 10.3390/ijerph110807642 25089772 PMC4143824

[53] Vrijheid M Martinez D Manzanares S Dadvand P Schembari A Rankin J Ambient air pollution and risk of congenital anomalies: a systematic review and meta-analysis. Environ Health Perspect. (2011) 119(5):598–606. 10.1289/ehp.1002946 21131253 PMC3094408

[54] Wondemagegn AT Afework M. The association between folic acid supplementation and congenital heart defects: systematic review and meta-analysis. SAGE Open Med. (2022) 10:20503121221081069. 10.1177/20503121221081069 35284077 PMC8905196

[55] Feng Y Wang S Chen R Tong X Wu Z Mo X. Maternal folic acid supplementation and the risk of congenital heart defects in offspring: a meta-analysis of epidemiological observational studies. Sci Rep. (2015) 5:8506. 10.1038/srep08506 25687545 PMC4330542

